# Longitudinal monitoring of *Gaussia* and Nano luciferase activities to concurrently assess ER calcium homeostasis and ER stress *in vivo*

**DOI:** 10.1371/journal.pone.0175481

**Published:** 2017-04-12

**Authors:** Emily S. Wires, Mark J. Henderson, Xiaokang Yan, Susanne Bäck, Kathleen A. Trychta, Molly H. Lutrey, Brandon K. Harvey

**Affiliations:** Intramural Research Program, National Institute on Drug Abuse, Baltimore, Maryland, United States of America; Duke University School of Medicine, UNITED STATES

## Abstract

The endoplasmic reticulum (ER) is essential to many cellular processes including protein processing, lipid metabolism and calcium storage. The ability to longitudinally monitor ER homeostasis in the same organism would offer insight into progressive molecular and cellular adaptations to physiologic or pathologic states, but has been challenging. We recently described the creation of a *Gaussia* luciferase (GLuc)-based secreted ER calcium-modulated protein (SERCaMP or GLuc-SERCaMP) to longitudinally monitor ER calcium homeostasis. Here we describe a complementary tool to measure the unfolded protein response (UPR), utilizing a UPRE-driven secreted Nano luciferase (UPRE-secNLuc) to examine the activating transcription factor-6 (ATF6) and inositol-requiring enzyme 1 (IRE1) pathways of the UPR. We observed an upregulation of endogenous ATF6- and XBP1-regulated genes following pharmacologically-induced ER stress that was consistent with responsiveness of the UPRE sensor. Both GLuc and NLuc-based reporters have favorable properties for *in vivo* studies, however, they are not easily used in combination due to overlapping substrate activities. We describe a method to measure the enzymatic activities of both reporters from a single sample and validated the approach using culture medium and rat blood samples to measure GLuc-SERCaMP and UPRE-secNLuc. Measuring GLuc and NLuc activities from the same sample allows for the robust and quantitative measurement of two cellular events or cell populations from a single biological sample. This study is the first to describe the *in vivo* measurement of UPRE activation by sampling blood, using an approach that allows concurrent interrogation of two components of ER homeostasis.

## Introduction

The endoplasmic reticulum (ER) is an essential component of the secretory pathway, serving as the main site for protein synthesis and modification, lipid metabolism, lipoprotein secretion, and calcium homeostasis [1]. Following perturbations, ER homeostasis is reestablished through the unfolded protein response (UPR). The UPR is an adaptive response employed by the ER to alleviate stress caused by misfolded proteins [2–4]. Endoplasmic reticulum stress is implicated in a variety of diseases and can be triggered by an array of stimuli [5, 6]. For example, the ER lumen contains the highest level of intracellular calcium, with concentrations estimated to be 1,000–10,000-fold greater than cytosolic levels [7]. Perturbations to this gradient can decrease chaperone activity and result in the initiation of the UPR. UPR activation is mediated by three transmembrane proteins; IRE1 (inositol-requiring protein-1), ATF6 (activating transcription factor- 6), and PERK (protein kinase RNA-like ER kinase). Under physiological conditions, these branches are sequestered within the ER membrane bound to GRP78 (glucose response protein-78 or BiP) [8]. Initial activation of the UPR increases expression of proteins that aid in protein folding, decreases general translation and translocates misfolded proteins to the cytosol for proteasomal degradation [3]. Inability to rectify ER stress, whether due to severity or prolonged activation, can result in cell death.

We previously developed and characterized a reporter protein for assessing ER calcium homeostasis where secretion of the reporter, GLuc-SERCaMP, occurs in response to depletion of ER calcium stores [9]. The temporal relationship between ER calcium depletion and the UPR is not fully understood, particularly in models of human disease where progressive changes in these cellular processes occur. Activation of the ATF6 and IRE1 arms of the UPR have been previously linked to ER calcium depletion. Thapsigargin (Tg), a pharmacological inhibitor of the sarco/endoplasmic calcium ATPase (SERCA) and depletor of luminal calcium, induces proteolytic cleavage and nuclear translocation of ATF6, which in turn activates ER stress response elements (ERSE) and unfolded protein response elements (UPRE) [10]. ER calcium depletion in rat cardiac myocytes results in ATF6 nuclear translocation, activation of the SERCA2 promoter and increases in SERCA2 protein levels [11]. These findings suggest ER calcium depletion and the ATF6 branch of the UPR are interconnected to restore ER calcium imbalance. Likewise, activation of the IRE1 branch increases following Tg treatment in keratinocyte and pancreatic cell lines [12, 13]. We sought to concurrently measure both GLuc-SERCaMP and UPR activation, from a single biological sample (e.g. blood), to delineate the roles of ER calcium homeostasis and the UPR. To complement the previously reported GLuc-SERCaMP reporter, we developed a secreted Nano luciferase (NLuc) reporter that is transcriptionally regulated by a UPRE sequence, which was previously demonstrated to be responsive to the ATF6 [14] and IRE1 [15] arms of the UPR.

NLuc is an attractive counterpart to GLuc for *in vivo* experiments due to its robust light emission and functional stability at human body temperature [16, 17], necessary characteristics for *in vivo* monitoring. Despite the advantages of NLuc and GLuc as individual reporters, this pair poses technological challenges that limit multiplexing. NLuc catalyzes the ATP-independent oxidation of furimazine (FMZ) to furimamide, yielding light as a product of the reaction [16]. Similarly, GLuc catalyzes the oxidation of coelenterazine (CTZ) to coelenteramide, also producing light in an ATP-independent reaction [18]. Assaying the enzymes in combination is limited primarily by two properties: 1) NLuc has substantial activity towards CTZ, an analog of FMZ [19]; and 2) the broad emission spectra are highly similar, with a maximum emission at 460 nm for NLuc and 480 nm for GLuc [16, 18]. Previous work focusing on a computational approach to discern luciferase activities from dual-transfected samples has been described, but is not amenable for longitudinal *in vivo* studies [19].

Here we describe an approach that separates GLuc and NLuc activities using enzyme-specific substrates to measure ER calcium dysregulation and UPR activation. The basis of this assay is the inclusion of a small molecule inhibitor of NLuc [20] such that it no longer reacts with CTZ, allowing for measurement of the individual enzymatic activities. We utilized this approach to develop a duplex assay for ER stress and ER calcium depletion. Current methodologies for monitoring UPR activation *in vivo* require *ex vivo* processing [21] or the use of transgenic models [22], both of which are useful but limited. The tools presented here expand upon preceding methods, allowing for longitudinal assessment of multiple components of ER homeostasis *in vivo*, and to our knowledge is the first report of its kind. The assays described enable the establishment of a temporal profile between ER calcium homeostasis and UPR activation in disease models and/or drug screening, and we envision this approach can be adapted to study other cellular pathways.

## Results

### Separately measuring the activities of GLuc and NLuc in a single biological sample

**Fig 1:**
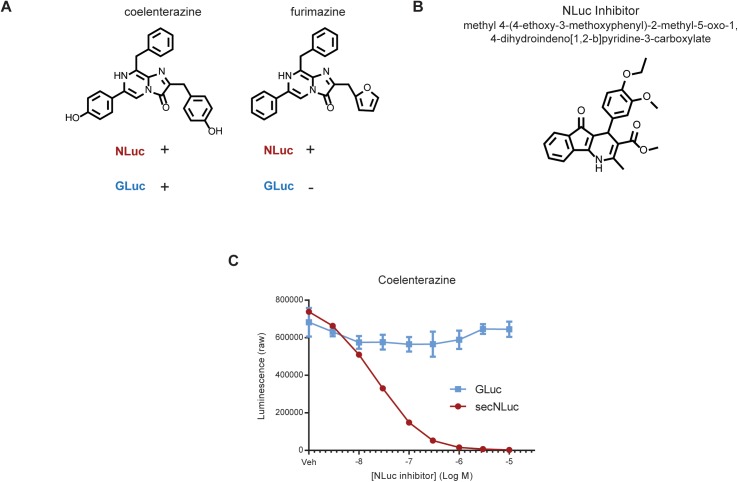
NLuc inhibitor blocks NLuc activity using CTZ as a substrate (A) Schematic showing expected activities of NLuc and GLuc with CTZ and FMZ substrates. (B) Chemical structure of the NLuc inhibitor. (C) The NLuc Inhibitor blocks the enzymatic activity of secNLuc, but not GLuc, towards CTZ. The inhibitor was added to 8 μM CTZ substrate and light emission was measured (mean ± SD, n = 3).

The NLuc inhibitor was previously identified in a high-throughput screen for compounds that would interfere with reporter gene assays (false positives) due to effects on NLuc/FMZ activity [20]. We hypothesized that this compound would also inhibit the activity of NLuc towards CTZ, thus allowing for measurement of GLuc and NLuc from a single sample. We first tested the enzymatic activity of GLuc and NLuc using two substrates, CTZ and FMZ (Fig 1A) in the presence methyl 4-(4-ethoxy-3-methoxyphenyl)-2-methyl-5-oxo-1,4-dihydroindeno[1,2-b] pyridine-3-carboxylate, the previously identified NLuc inhibitor (referred to as “NLuc inhibitor” hereafter (Fig 1B). The enzymatic activity of GLuc and NLuc on CTZ was measured in medium collected from SH-SY5Y human neuroblastoma cells expressing GLuc or secNLuc, a NLuc modified to be secreted. As expected, GLuc and secNLuc both showed activity towards CTZ, with GLuc exhibiting flash kinetics and secNLuc producing a stable light emission profile (S1 Fig). The NLuc inhibitor decreased enzymatic activity of secNLuc towards CTZ, in a dose dependent manner, with minimal effect on GLuc activity (Fig 1C). secNLuc showed stable light emission with its substrate FMZ, whereas GLuc had no detectable activity towards FMZ (S1 Fig). We observed a linear relationship between light emission and enzyme concentration for secNLuc/FMZ and GLuc/CTZ+inhibitor (S1 Fig).

For these studies, FMZ substrate was prepared according to the manufacturer’s instructions followed by a 20-fold dilution in PBS, as sufficient signal intensity and decay kinetics were observed under these conditions (S2 Fig). Raw luminescence values increased with use of diluted substrate, which we hypothesize is due to the presence of an inhibitor that extends the light emission profile at the expense of brightness in the commercial substrate buffer. In line with this hypothesis, we observed a more rapid decay of luminescence over twenty minutes when substrate was diluted 20-fold in PBS (S2 Fig). For our experiments, however, decay at the 20-fold dilution was not a concern due to the short time interval required to add substrate to all samples within an experiment (less than 1 minute). For experiments where a significant time is elapsed between addition of substrate to the first and last sample, the assay buffer composition can be adjusted to circumvent signal decay.

### GLuc-SERCaMP and secNLuc dual-reporter assay to monitor ER calcium homeostasis

**Fig 2:**
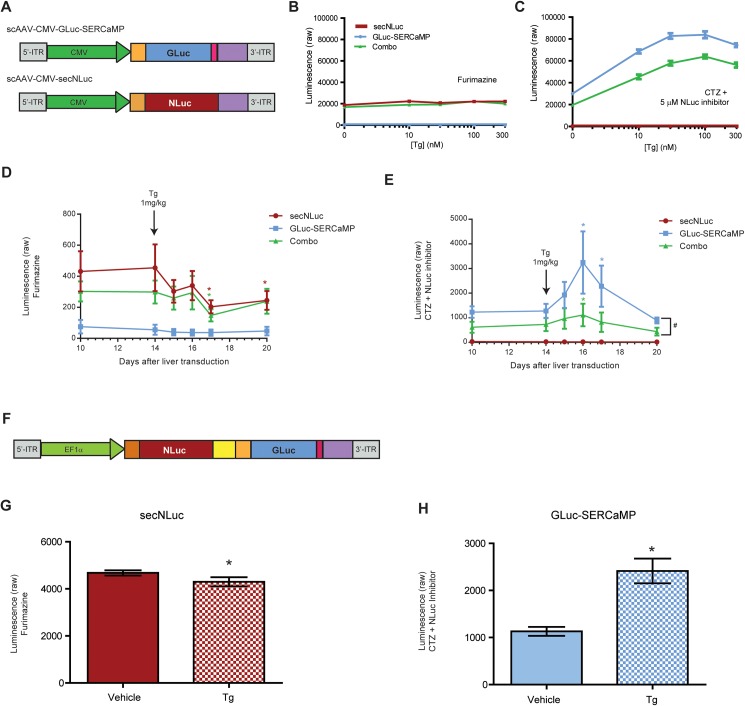
Dual luciferase assay can be used to examine ER-calcium-dependent (GLuc-SERCaMP) and constitutive (secNLuc) secretion. (A) Schematic of AAV-constructs used to express secNLuc and GLuc-SERCaMP. Orange represents hMANF signal peptide, pink is hMANF C-terminal peptide, and purple is SV40 polyA tail. (B, C) Constitutive secretion and ER calcium-dependent secretion can be monitored in rat primary cortical neurons using the dual GLuc and secNLuc reporter approach. Neurons were transduced with AAV-secNLuc, AAV-GLuc-SERCaMP, or a combination of both viruses. Secreted levels of the reporter proteins were assessed after 8 hrs of Tg (0–300 nM) treatment using 5 μL of culture medium and 100 μL of the indicated substrate (mean ± SEM, n = 12). (D, E) Blood was collected from rats expressing AAV-secNLuc, AAV-GLuc-SERCaMP, or a combination of both, prior to and following 1 mg/kg Tg exposure. Plasma was assayed for GLuc-SERCaMP or secNLuc using CTZ plus NLuc inhibitor or FMZ, respectively. Raw luminescence values are shown (mean ± SEM, n = 4 per group, 2-way ANOVA, * p<0.05, Dunnett’s multiple comparison test vs. day 14; # p<0.05 Tukey’s multiple comparison test between viral groups). (F) Design of viral construct expressing both secNLuc and GLuc-SERCaMP from a single transgene using a self-cleaving 2A peptide. (G, H) SH-SY5Y cells transfected with pAAV-EF1α-secNLuc-2A-GLuc- SERCaMP were treated with 300 nM Tg for 8 hrs. (G) Medium was collected and assayed for secNLuc using FMZ (mean ± SD, n = 6; *p<0.01, two-tailed t-test). (H) Medium was collected and assayed for GLuc-SERCaMP activity using 8 μM CTZ + 5 μM NLuc inhibitor (mean ± SD, n = 6; *p<0.01, two-tailed t- test).

A set of adeno-associated viral (AAV) vectors (serotype 1) was constructed to express secNLuc and GLuc-SERCaMP (Fig 2A). Using these in combination allows for assessment of ER calcium homeostasis (GLuc-SERCaMP) [9], while controlling for general effects on the secretory pathway (constitutively secreted NLuc), from the same sample. Rat primary cortical neurons were transduced with AAV-secNLuc, AAV-GLuc-SERCaMP, or a combination of the viruses. Medium was collected after treatment with Tg, a SERCA pump inhibitor used to deplete the ER calcium store. As expected, only GLuc-SERCaMP secretion was altered by thapsigargin administration (Fig 2B and 2C). Additionally, only cells expressing secNLuc showed activity towards FMZ (Fig 2B) and those expressing GLuc-SERCaMP towards CTZ plus inhibitor (Fig 2C).

The utility of measuring GLuc and secNLuc activities in a single biological sample may be most advantageous for *in vivo* experiments, where multiple cell populations or biological events can be tracked in a single animal over time. Here we conducted *in vivo* experiments using a previously described liver model of ER stress [9, 23] to measure GLuc and secNLuc enzymatic activities in plasma from a single animal. Rats were injected intrahepatically with AAV-GLuc-SERCaMP, AAV-secNLuc, or a combination of the viruses (Fig 2D and 2E). First, the efficacy of the NLuc inhibitor was assessed in plasma samples. We observed a 99% reduction of secNLuc light emission at 5 μM inhibitor (S1 Fig). Furthermore, the inhibitor showed minimal effect on GLuc enzymatic activity in plasma under identical conditions (S1 Fig). Fourteen days after intrahepatic injection of AAV-secNLuc, AAV-GLuc-SERCaMP, or a combination of viruses, rats were given 1 mg/kg intraperitoneal injection of Tg to cause systemic disruption of ER calcium. We have previously demonstrated this approach to be effective in triggering release of GLuc-SERCaMP from rat liver [9]. Blood samples were collected before and after Tg administration, and enzymatic activity was assessed using the two-substrate approach. The secNLuc reporter activity in the plasma was decreased following Tg treatment indicating a drop in constitutive secretion, whereas GLuc-SERCaMP activity in the plasma increased following Tg. This trend was observed when virus was administered alone or together. The combination of viruses showed a diminished magnitude of response, but similar trends in activity for each of the reporters (Fig 2D and 2E).

To create a single viral vector expressing the two luciferase reporters, we constructed a plasmid encoding both secNLuc and GLuc-SERCaMP (Fig 2F). The design utilized a single promoter with the transgenes separated by a foot-and-mouth disease virus-derived 2A peptide cleavage sequence [24] and a Ser-Gly-Ser-Gly spacer [25]. Enzymatic activity of the two transgenes in SH-SY5Y cells treated with Tg was examined using FMZ (Fig 2G) and CTZ plus NLuc inhibitor (Fig 2H). GLuc-SERCaMP activity increased in response to Tg (Fig 2H) whereas secNLuc slightly decreased in response to Tg (Fig 2G), a result consistent with delivering the reporters as independent vectors (Fig 2B and 2C).

### UPRE-dependent secNLuc reporter for monitoring the UPR

**Fig 3:**
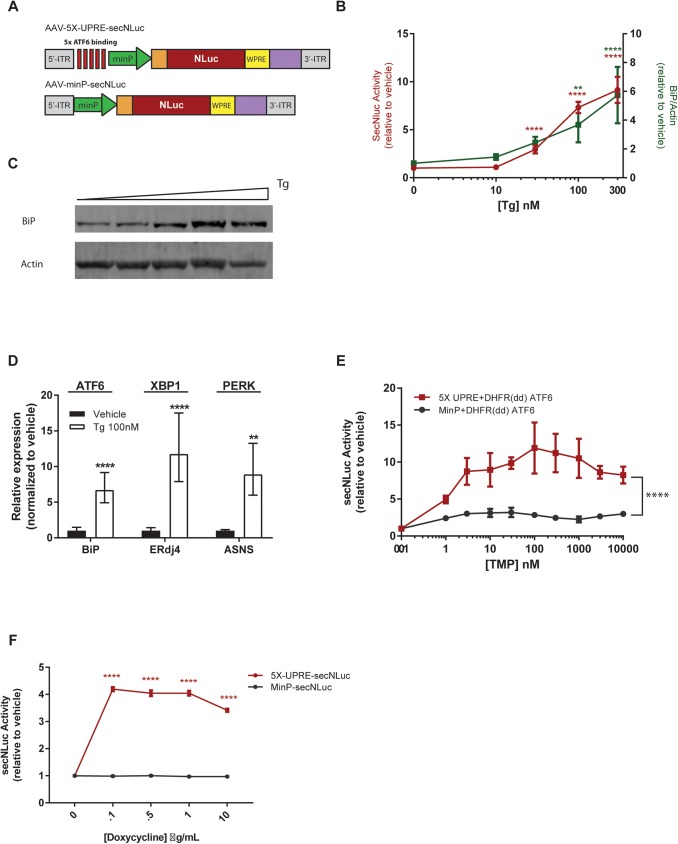
UPRE-induced secNLuc can be used to monitor ER stress. (A) Schematic of AAV constructs used to express UPRE-dependent and control (minimal promoter, minP) secNLuc reporters. Tg treatment (100 nM) increases (B) 5X-UPRE-secNLuc activity (culture medium, 24 hrs, mean ± SD, n = 9) and endogenous BiP protein levels in the cell (cell lysates, 24 hrs, mean ± SD, n = 4). (C) Representative blot of BiP/actin as function of Tg treatment used for graph in (B). (D) Expression levels of UPR-responsive genes BiP, ERdj4, and ASNS (mRNA analysis, 8 hrs, mean ± SD n = 9) **p<0.01, ****p<0.0001, 1-way ANOVA, Dunnett’s multiple comparison test vehicle vs Tg). (E) TMP induces 5X-UPRE-secNLuc but has minimal effect on MinP-secNLuc (mean ± SD, n = 6 wells/transfection/TMP treatment ****p<0.0001, 2-way ANOVA). (F) Doxycycline-inducible XBP1 activates 5X-UPRE-secNLuc in HEK^DAX^ cells (mean ± SEM, n = 4 wells/transfection/doxycycline treatment ****p<0.0001, 2-way ANOVA, Sidak’s multiple comparison test).

The UPR is an adaptive mechanism employed by cells to alleviate ER stress. Here we designed an approach to track transcriptional activity of ATF6 and XBP1 to longitudinally monitor UPR. We created a reporter, 5X-UPRE-secNLuc, containing five UPRE binding sites followed by a minimal promoter to drive the expression of secNLuc (Fig 3A). SH-SY5Y cells transfected with 5X-UPRE-secNLuc showed a dose-dependent increase in secNLuc activity following Tg treatment, which tracked well with an increase in BiP protein (Fig 3B and 3C), as well as with mRNA levels of *BiP*, *ERdj4*, and *ASNS* (asparagine synthetase), endogenous UPR-responsive genes activated mainly by the ATF6, IRE1/XBP1, or PERK signaling cascades, respectively [26, 27](Fig 3D). The control reporter, MinP-secNLuc, which lacks the 5X UPRE binding sites showed minimal response to Tg (S3 Fig). To confirm 5X-UPRE-secNLuc expression is regulated by ATF6 activation, a chemically-inducible model of ATF6 signaling was used [26]. SH-SY5Y cells were co-transfected with 5X-UPRE-secNLuc reporter and DHFR(dd) ATF6, a destabilized form of the protein. Briefly, when ATF6 is fused to a destabilizing domain (DHFRdd), ATF6 is unstable and constitutively degraded. However, upon the addition of trimethoprim (TMP), a pharmacological stabilizer of DFHR, ATF6 becomes stabilized and is a functional activator of ATF6-dependent genes [26]. Cells co-transfected with AAV-5X-UPRE-secNLuc and DHFR(dd) ATF6 showed a TMP dose-dependent increase in secNLuc activity, whereas TMP-stabilized ATF6 had a minimal effect on MinP-secNLuc control (Fig 3E). To confirm XBP1 can also activate 5X-UPRE-secNLuc, we used the previously described cell line, HEK293^DAX^ [26], to chemically induce XBP1. Cells transfected with 5X-UPRE-secNLuc displayed increased luciferase activity in the presence of doxycycline, whereas the MinP-secNLuc was unresponsive (Fig 3F). These data are the first to demonstrate a secreted luciferase-based monitoring system of UPRE activation and indicate that UPR activation can be monitored by sampling extracellular fluid. TMP- or doxycycline-mediated activation of the ATF6 and XBP1 pathways in the HEK293^DAX^ cell line was confirmed by analysis of *BiP* and *ERdj4* transcript levels (S3 Fig).

### Concurrently monitoring the UPR and ER calcium homeostasis using UPRE-secNLuc and GLuc-SERCaMP

**Fig 4:**
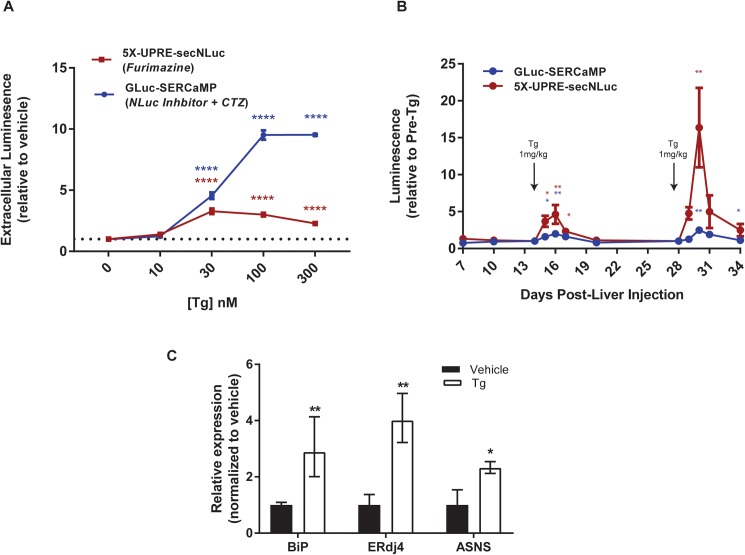
Dual luciferase assay to examine ER calcium dysregulation and UPRE-induced secNLuc *in vitro* and *in vivo*. (A) Concurrent measurement of ER calcium homeostasis and ER stress by measuring extracellular luciferase in medium. SH-SY5Y-GLuc-SERCaMP stable cells were transfected with 5X-UPRE-secNLuc and treated with Tg (0–300 nM)) for 8 hrs (mean ± SEM, n = 4 wells/transfection/ treatment, *p<0.05, **p<0.01, ****p<0.0001, 2-way ANOVA, Dunnett’s multiple comparison test vs vehicle). (B) Effect of Tg on plasma levels of GLuc and secNLuc. Rats were intrahepatically injected with AAV-GLuc-SERCaMP and AAV-5X-UPRE-secNLuc. Luminescence was normalized to levels measured immediately prior to Tg injection (mean ± SEM, n = 4 rats, 1-way ANOVA, Dunnett’s multiple comparison test vs. Tg administration (days 14 and 28), *p<0.05, **p<0.01). (C) RT-qPCR analysis of UPR targets, *BiP*, *Erjd4*, and *ASNS*, from rat liver tissue 24 hrs post-Tg (1 mg/kg) or vehicle administration. Data expressed as fold change (2^-ΔΔCt^) relative to vehicle-treated rats (mean ± upper and lower limits (2^-ΔΔCt^±SD), n = 3 rats (Tg), n = 4 rats (vehicle), unpaired t-test (Tg vs vehicle) transformed from ΔCt values, *p<0.05, **p<0.01).

Using the dual reporter secNLuc and GLuc assay, we proceeded to investigate both ER calcium homeostasis and the UPR in a single organism over time. To validate this approach *in vitro*, a SH-SY5Y GLuc-SERCaMP stable cell line previously described [9] was transfected with the 5X-UPRE-secNLuc and treated with Tg, tunicamycin (TM) or brefeldin A (BFA). The extracellular luciferase activity of both sensors increased in a dose-dependent manner in response to Tg treatment (Fig 4A). Tunicamycin and BFA had a minimal effect on extracellular levels of both GLuc-SERCaMP and secNLuc (S4 Fig) and robustly increased intracellular levels of secNLuc (S4 Fig) confirming the 5X-UPRE-secNLuc is strongly activated by ER stress. Brefeldin A can directly inhibit secretion by blocking vesicle formation and TM indirectly impairs secretion via inhibition of N-linked protein glycosylation of secretory proteins. These results highlight the need for appropriate controls to monitor protein secretion, such as a coexpressed and constitutively secreted GLuc in place of GLuc-SERCaMP [9], when evaluating an ER stress response.

To investigate the utility of the dual reporter approach for measuring ER calcium and ER stress *in vivo*, we again employed the liver model of ER stress. Animals were intrahepatically co-injected with AAV-GLuc-SERCaMP and AAV-5X-UPRE-secNLuc into the right medial lobe and administered Tg (1 mg/kg) intraperitoneally at day 14 and 28 (Fig 4B). Following each Tg injection, NLuc and GLuc activities were significantly increased when compared to pre-Tg levels and notably returned to pre-Tg levels after several days (Fig 4B). To confirm the UPRE reporter activation was biologically relevant, we assessed the endogenous UPR by measuring the mRNA levels of *BiP*, *Erdj4*, and *ASNS* in liver. Twenty-four hrs post-Tg, *BiP*, *Erdj4*, and *ASNS* mRNA levels increased by 2.9, 4.0, and 2.3-fold, respectively when compared to vehicle-treated rats (Fig 4C). Upregulation of these endogenous genes is consistent with UPR activation in the liver model system employed in these experiments (Fig 4B).

## Discussion

The ability to longitudinally monitor biological process in the same organism over time can offer insight into the progressive molecular and cellular adaptations to different physiologic or pathologic states. The therapeutic efficacy of drugs targeting these biological processes, in the context of disease, could also be monitored longitudinally. Here we demonstrate the ability to measure two robust bioluminescent protein-based reporters in extracellular fluid as a general platform to longitudinally monitor biological processes. More specifically, we demonstrate the ability to concurrently measure ER calcium homeostasis and UPRE activation over time in rat liver.

Dual-enzymatic based assays have been available for over 20 years, allowing for measurement of two biological events or providing internal controls for experiments [28]. Our work to expand this potential to the NLuc and GLuc pair, which have higher signal-to-noise and more robust luminescence than predecessors [16, 18] allows for new experimental opportunities, particularly *in vivo*. Use of an NLuc inhibitor to separate enzymatic activities provides a simple approach to multiplexing the enzymes. Previous work described a dual luciferase assay to measure NLuc and GLuc activity based on mathematical extrapolation instead of direct measurement [19], however there were caveats to this approach. Using methyl 4-(4-ethoxy-3-methoxyphenyl)-2-methyl-5-oxo-1,4-dihydroindeno[1,2-b] pyridine-3-carboxylate [20], to inhibit ~99% of NLuc activity we were able to isolate GLuc activity. Recognizing that there is low residual NLuc activity (less than 1%) in presence of the inhibitor can be used to guide transgene design. For instance, if one transgene is expected to have several orders of magnitude greater expression, GLuc should be used for the higher expressing of the pair and NLuc for the lower expressing. This reduces the risk of NLuc cross-reactivity impacting the ability to effectively separate activities.

ER calcium is imperative to cellular functions and its dysregulation is associated with a wide variety of prominent human diseases including diabetes, stroke, cardiac disease and asthma [29]. We previously identified a protein modification that creates a secreted, ER calcium modulated protein (SERCaMP) which was used to make a GLuc-based reporter, GLuc-SERCaMP [9]. The ability to repeatedly sample blood from an animal or a small volume of medium from the same cell culture vessel, without the need to lyse cells to detect enzymatic activity, is also advantageous for establishing temporal relationship with cellular processes. We sought a means to measure a constitutively secreted protein, to control for effects unrelated to ER calcium depletion, and thus created a dual reporter utilizing secNLuc (Fig 2). These experiments demonstrated Tg-induced GLuc-SERCaMP release from rat primary neurons (*in vitro*) and rat liver (*in vivo*) was not due to global increases in protein secretion. Notably, we observed a Tg-induced increase in circulating GLuc-SERCaMP with a corresponding decrease in circulating levels of constitutively secreted NLuc (Fig 2D and 2E). These data are consistent with GLuc-SERCaMP as a reporter of ER calcium dysregulation. We observed the GLuc-SERCaMP response was smaller in magnitude when liver was transduced with the combination of viruses compared to GLuc-SERCaMP alone. The discrepancy observed in co-injected rats is likely due to sub-optimal viral delivery parameters pertaining to viral concentration and/or viral receptor competition. The total number of viral genomes injected differed between single injection versus the combined virus injection (50% of viral particles for the combination injection) which resulted in about 50% expression. In regards to receptor competition, both viruses were AAV serotype 1. AAV1, in particular, binds to sialic acids present on N-linked glycoproteins [30]. Wu et al., 2006 demonstrated compromised AAV1 transduction efficiency in the presence of a 200-fold excess empty AAV1 vector 1. The same competition effect would be predicted for Fig 4B, where both viruses were AAV serotype 1 and AAV-5X-UPRE-secNLuc was injected at a 710-fold excess compared to GLuc-SERCaMP. Although our data provide a working titer for the indicated reporters in rat liver, optimization of reporter transgene delivery will be necessary for each experimental paradigm (e.g. virus type, reporter design, target tissue).

The aforementioned ability to discern GLuc and NLuc activities was further exploited to measure ER calcium homeostasis and UPR activation. We developed and characterized a UPRE-dependent secNLuc reporter to measure UPRE activation in response to ER stressors, Tg, TM, and BFA. However, the mechanistic nature of the stressor must be taken into consideration when using a secreted reporter, as ER stress can hinder the secretory pathway. For example, both BFA and TM negatively impact protein secretion directly via inhibition of vesicle formation and indirectly via inhibition of N-linked protein glycosylation of secretory proteins, respectively. BFA caused a slight decrease in extracellular luciferase activity for the 5X-UPRE-secNLuc reporter and TM caused a minimal increase (1.2 fold) in extracellular luciferase activity only at the highest concentration. However, we observed a robust increase in the intracellular levels of secNLuc, consistent with a strong activation of ATF6/XBP1 and subsequent inability of secNLuc to traverse the secretory pathway. These results highlight the need to employ appropriate controls for effects on general secretion, such as shown in Fig 2, to interpret UPRE-secNLuc results. In the case where general secretion is affected, ATF6/XBP1 activation and ER calcium homeostasis can be evaluated by using single endpoint analysis to measure intracellular levels of the reporters as well. Alternatively, using the methods described in this paper, GLuc without the SERCaMP modification [9] could be combined with 5X-UPRE-secNLuc to examine ER stress and protein secretion in parallel *in vitro* or *in vivo*.

The ability of the UPRE-secNLuc reporter to reflect endogenous UPR activity was examined using both *in vitro* and *in vivo* models, where we examined ATF6- and XBP1-regulated genes, *BiP/GRP78 and ERdj4* [26]. The ability of both the ATF6 and IRE1 arms to activate the reporter was confirmed using a ligand-inducible form of ATF6 and a doxycycline inducible form of XBP1 [10, 26]. *In vivo*, ER calcium homeostasis and UPRE activation were monitored over time, and both were responsive to Tg administration. This is the first demonstration of concurrently measuring the UPR and ER calcium homeostasis in the same animal over time by repeated sampling of blood. Examining this temporal profile is not only unique, but crucial to further elucidate the role of ER calcium and the UPR in rodent models of disease. Optimizing the co-delivery of these sensors will be required for each target tissue and disease model to be examined. Overall, our results demonstrate that it is possible to measure both GLuc and secNLuc activities in rat plasma, opening opportunities to use this approach to probe two biological processes, such as ER calcium homeostasis and the UPR.

## Materials and methods

### Plasmid construction

GLuc-SERCaMP plasmid construction has been previously described [9]. The pAAV-5XUPRE-secNLuc plasmid (Addgene #82497) was constructed using p5X ATF6-GL3 (Addgene #11976) as a template to amplify the five UPRE binding sites and c-fos minimal promoter region [14] using the following primers: (fwd)-5’- ctgcggccgcacgcgttgcagcccaagcttgctcg -3’ and (rev)- 5’-ggccgccggctcagt-3’. For the control plasmid, pAAV minP-secNLuc (Addgene #82498), the c-fos minimal promoter region (lacking the 5xATF6 binding sites) was amplified with (fwd)-5’- ctgcggccgcacgcgtcactcattcataaaacgcttgttataaaagc-3’ and (rev)- 5’-ggccgccggctcagt-3’. The secNLuc coding sequence was amplified from pNL1.3 (Promega, Madison, WI) using the following primers: (fwd)-5’- actgagccggcggccggtactgttggtaaagccaccatg-3’ and (rev)-5’- atcgaattcggcgcgccactctagagtcgcggcctta-3’. Phusion high-fidelity DNA polymerase (New England Biolabs) was used for all PCR reactions. For pAAV-EF1a-secNLuc-2A-GLuc-SERCaMP (Addgene #68367), the coding region was cloned into the NheI and AscI sites of pOTTC809. A three-piece In-Fusion HD Cloning reaction was performed using secNLuc (amplified from pNL1.3, adding a Kozak sequence upstream of AUG), a Gblock encoding the SSGG-spacer followed by a 2A cleavage site (IDT DNA Technologies), and GLuc-SERCaMP (amplified from pdsAAV-CMV-GLuc-SERCaMP)[9]. PCR products were gel purified and the In-Fusion reaction contained 100 fmol of each fragment and 50 fmol of the backbone. pdsAAV-CMV-secNLuc was created using pdsAAV-CMV-GLuc-SERCaMP plasmid and pNL1.3 (Promega) as templates. GLuc-SERCaMP and secNLuc coding regions were removed from respective plasmids using restriction enzymes, Kpn1 and Xba1 (New England BioLabs). secNLuc was ligated within the linearized pdsAAV-CMV-GLuc-SERCaMP backbone. All plasmids were sequence verified. HEK293^DAX^ cells and pLenti CMV DHFR(dd)-hATF6 was a generous gift from Dr. Matthew Shoulders [26].

### Virus production

All vectors were prepared using triple transfection method in HEK293 cells as previously described [31]. Cell and media pellets were thawed and then combined for a series of two freeze-thaw cycles. MgCl_2_ (2 mM final; Sigma-Aldrich, St. Louis, MO) and Benzonase (EMD Millipore, Billerica, MA) were added at 50 U/ml of cell solution for 1 hr at 37°C while shaking. The mixture was then centrifuged for 20 min at 2450 × *g* at 4°C. Supernatant was transferred to 75 mL PBS with 2 mM MgCl_2_ and sequentially filtered through 5-, 0.45-, and 0.22 μm filters followed by a pass through of 1 mL AVB Sepharose HI-TRAP column (GE Healthcare, Pittsburg, PA) using an AKTA purifier (GE Healthcare) at a rate of 2 mL/min and eluted using a 15 mM sodium citrate solution (Sigma-Aldrich) at a rate of 1 mL/min. The fractions containing the peak of the OD_254_ and OD_280_ readings were collected and dialyzed using a 10,000 MWCO dialysis cassette (Thermo Fisher Scientific, Waltham, MA) in 1 L of PBS containing 0.5 mM MgCl_2_ for three exchanges over 25–30 hrs. The equilibrated virus was aliquoted, snap-frozen, and stored at −80°C.

### Cell culture

SH-SY5Y-GLuc-SERCaMP and SH-SY5Y cells were cultured in a 37°C humidified incubator with 5.5% CO_2_ in DMEM (4.5 g/L D-glucose) containing 2 mM GlutaMAX, 10% bovine growth serum (Sigma Aldrich), 10 U/mL penicillin (Thermo Fisher Scientific), and 10 μg/mL streptomycin (Thermo Fisher Scientific). Cells were plated at 5 x 10^4^ cells per well (100 μL volume). Cells were incubated for 8 or 24 hrs prior to adding 100 nM Tg (Sigma-Aldrich). Media was collected (5 μL) prior to and at indicated time points post-drug treatment for enzymatic assay as previously described [23]. Intracellular NLuc was assessed by washing cells with 1X PBS and lysing in Tris-HCl, 105 mM NaCl, 1%5 NP-40 and 1X protease inhibitor. For TMP experiments, SH-SY5Y cells were plated at 1.75x10^5^ cells per T25 flask. Cells were co-transfected with 2 μg of pAAV-5X-UPRE-secNLuc or pAAV-minP-secNLuc and 8 μg of pCMV-DHFR(dd)-hATF6 using Lipofectamine 2000 (Thermo Fisher Scientific), as per manufacturers protocol. Three hours later transfection mixture was removed and replaced with cell culture media. Cells were incubated at 37°C for 36–48 hrs and replated into 96 well plates at 5x10^4^ per well, in a total volume of 100μl. Cells were treated with TMP (Sigma-Aldrich) for 16 hrs. For the drug (Tg, TM and BFA) dose response experiments, the SH-SY5Y-GLuc-SERCaMP cells were plated and transfected as in TMP experiments then treated Tg (Sigma-Aldrich), TM (Sigma-Aldrich) and BFA (Sigma-Aldrich) at 18 hrs after replating at indicated concentrations and remained on cells for 8 hrs. Isolation and maintenance of rat primary cortical cultures have been previously described [31].

HEK^DAX^ cells were cultured in a 37°C humidified incubator with 5.5% CO_2_ in DMEM (4.5 g/L D-glucose) containing 2mM GlutaMAX, 10% fetal bovine serum (Sigma Aldrich), 10 U/mL penicillin (Thermo Fisher Scientific), and 10 μg/mL streptomycin (Thermo Fisher Scientific). Cells were plated at 3.5x10^6^ cells per T-25 flask. Cells were transfected with 5 μg of pAAV-5X-UPRE-secNLuc or pAAV-minP-secNLuc using Lipofectamine 2000 (Thermo Fisher Scientific), as per manufacturers protocol. Three hours later, the transfection mixture was removed and replaced with cell culture media. Cells were incubated at 37°C for 36 hrs and replated into 96 well plates at 5x10^4^ per well, in a total volume of 100μl. Doxycycline (Sigma-Aldrich) was added 18 hrs later at the indicated concentrations and remained on cells for 16 hrs. For RNA isolation, 2.5x10^5^ cells/well were plated on a 24-well plate, and approximately 24 hrs later cells were treated with 10 μMTMP and/or 1 μg/mL doxycycline for 16 hrs.

### *Gaussia* luciferase secretion assay

Five microliters of culture medium (*in vitro*) or 10 μL of plasma (*in vivo*) was transferred to white 96-well plates. CTZ (Regis Technologies, Morton Grove, IL) was prepared as previously described [9, 23], however, it is worth noting, final concentrations of CTZ for *in vitro* and *in vivo* were 10 μM and 100 μM, respectively. Prepared substrate was incubated at room temperature 30 mins prior to use. NLuc inhibitor, methyl 4-(4-ethoxy-3-methoxyphenyl)-2-methyl-5-oxo-1,4-dihydroindeno[1,2-b] pyridine-3-carboxylate (MCule #5781513678, Palo Alto, CA) was added to a final concentration of 5 μM. One hundred microliters of substrate was injected into each well followed by immediate luminescence reading. Plate reader parameters for *in vitro* and *in vivo* samples included an integration time of .5 secs and a sensitivity of 100 and 5 secs and a sensitivity of 150, respectively (Biotek Synergy II, Winooski, VT). For NLuc readings, the same volumes of culture medium and plasma were transferred to opaque-walled plates, FMZ (Promega) was prepared according to the manufacturer’s protocol and then diluted 20-fold and 10-fold in 1X PBS for culture media and plasma, respectively. Wells were read on individual basis with same plate reader parameters as described above.

### Western blot

SH-SY5Y cells were plated at 7.5 x 10^5^ cells per well (1.5 mL volume) in 12 well plates and treated with DMSO or Tg (Sigma-Aldrich, St. Louis, MO). Cells were rinsed in PBS and a modified RIPA buffer containing 50 mM Tris-HCl (pH 7.4), 150 mM NaCl, 1 mM EDTA, 1% Nonidet P-40, and 1X protease inhibitor was added to each well. Plates were rotated for at least 20 mins at 4°C. Lysates were centrifuged for 10 mins at 13,000 x g (4°C) and quantified using a DC assay (Bio-Rad Laboratories, Hercules, CA) and equal amounts of total protein were loaded on 4–12% NuPAGE gels with MOPS running buffer (Thermo Fisher Scientific). Proteins were transferred to 0.45 μm polyvinylidene fluoride membranes (Life Technologies) and immunoblotted with the primary antibodies rabbit Anti-BiP (Cell Signaling, Danvers, MA) and mouse Anti-Actin (Abcam, Cambridge UK). Secondary antibodies were IR700 and IR800 (Rockland Immunochemicals, Gilbertsville, PA), and blots were scanned using an Odyssey scanner (LI-COR).

### RT-qPCR

mRNA expression was measured by real time RT-qPCR. Total RNA was isolated from rat liver tissue (right medial lobe) using the RNeasy Lipid Tissue Mini Kit together with the RNase-free DNase set (Qiagen, Germantown, MD) according to the instructions of the manufacturer. Total RNA from cell samples was isolated using the NucleoSpin RNA kit (Macherey-Nagel GmbH & Co., Düren, Germany). The RNA concentration was quantified with NanoDrop spectrophotometer (Thermo Fisher Scientific), and RNA samples were stored at -80°C until cDNA synthesis. Using the iScript cDNA Synthesis Kit (Bio-Rad Laboratories), 1 μg of the RNA samples was transcribed into cDNA in a 20μl reaction mix according to the instructions in the kit, and diluted 1:20 with DNase-free water. Primers and probes (S1 Table) were ordered from Integrated DNA Technologies (Coralville, IA). cDNA, along with appropriate primer/probe sets (final concentration 450 nM and 50 nM, respectively), and 2X Universal TaqMan Master Mix (Thermo Fisher Scientific) were amplified in a 20μl reaction mix using BioRad CFX96 (5 min at 95°C, 50 cycles of 20 secs at 94°C, 1min at 60°C). Ct values were normalized to the housekeeping gene Ube2i [32]. Fold change was calculated using 2^-ΔΔCt^ and data expressed as fold change with upper and lower limits (2^-ΔΔCt^±SD).

### Intrahepatic injection of AAV-based reporters

The animal studies conducted within this manuscript were approved by the NIDA IACUC and comply with the guidelines for animal research set by the National Institutes of Health. GLuc-SERCaMP intrahepatic injections of male Sprague Dawley rats have been previously described [9, 23], and in these experiments virus was injected at 7.6 x 10^9^ vg/mL. AAV1-5X-UPRE-secNLuc was intrahepatically injected at 4.16 x 10^11^vg/ml. AAV-CMV-secNLuc was injected at 7.6 x 10^9^ vg/mL. Injection volume did not exceed 105 μL (injected at 3 sites using approximately 33 μL per site). Blood collection and Tg administration have been previously described [9, 23].

## Supporting information

S1 Fig Development of approach to measure GLuc and secNLuc from biological samples containing both proteins. A) secNLuc and GLuc both show activity with coelenterazine substrate. Culture medium was collected from SH-SY5Y cells transfected with secNLuc or GLuc after 48 hrs. Light emission was monitored over 10 min after adding 8μM coelenterazine (mean ± SD, n = 2). (B) Kinetic analysis of secNLuc and GLuc light emission using 8μM coelenterazine containing 5μM NLuc inhibitor (mean ± SD, n = 2) (C) Kinetic analysis of secNLuc and GLuc light emission with 1X furimazine substrate (mean ± SD, n = 2). (D) Linear relationship is observed for secNLuc concentration versus light emission for 0.05X furimazine substrate. Medium was collected from SH-SY5Y cells transfected with secNLuc and diluted with medium from untransfected SH-SY5Y cells. Light emission was measured (mean ± SD, n = 4). (E) Linear relationship is observed for GLuc concentration versus light emission using coelenterazine substrate (8μM). Medium was collect emission using coelenterazine substrate (8μM). Medium was collected from SH-SY5Y cells transfected with GLuc and diluted with medium from untransfected SH-SY5Y cells. Light emission was measured (mean ± SD, n = 3) (F) NLuc inhibitor has activity on secNLuc, but not GLuc in plasma. NLuc inhibitor at various concentrations was added to coelenterazine substrate (100μM final) and light emission was measured in plasma containing GLuc-SERCaMP or secNLuc (mean ± SD, n = 3). elenterazine substrate (100μM final) and light emission was measured in plasma containing GLuc-SERCaMP or secNLuc (mean ± SD, n = 3).
(PDF)

S2 Fig Diluting furimazine substrate in PBS provides sufficient signal and acceptable light emission kinetics. Medium was collected from SH-SY5Y cells transiently transfected with scAAV-CMV-secNLuc plasmid. 100 μl of furimazine substrate, diluted as indicated in the figure inset, was added to 5μl of culture medium and luminescence was measured every 20 sec over 20 min. (A) Raw luminescence values (mean ± SD, n = 3). (B) Luminescence values were normalized to time zero to assess decay kinetics over the time course (mean ± SD, n = 3).
(PDF)

S3 Fig Further characterization of 5X-UPRE-secNLuc constructs. (A) SH-SY5Y cells transfected with 5x-UPRE-secNLuc or MinP-secNLuc and treated with Tg (0-300nM) for 24 hrs (mean ± SD, n = 12 wells/Tg treatment, ****p<0.0001, 2-way ANOVA).(B) ATP viability of SH-SY5Y cells transfected with 5X-UPRE-secNLuc or MinP-secNLuc and treated with Tg (0-300nM) for 24 hrs (mean ± SD, n = 12 wells/Tg treatment) (C) SY5Y cells transfected with 5X-UPRE-secNLuc or MinP-secNLuc and treated with 100 nM thapsigargin for indicated time (mean ± SEM, n = 6 wells/timepoint ***p<0.001, ****p<0.0001, 2-way ANOVA, Sidak’s multiple comparison test). D) Relative expression of BiP and ERdj4 after 16 hrs activation of HFR.ATF6 with 10 μM trimethoprim (TMP) and/or XBP1 with1 μg/mL doxycycline (DOX) (mean ± upper and lower limits (2^-ΔΔCt±SD), n = 6, ****p<0.0001 as compared to control, ####p<0.0001 as compared to DOX only, %%%p<0.001 as compared to TMP only, one-way ANOVA, Tukey’s multiple comparison test).
(PDF)

S4 Fig Dual luciferase assay using brefeldin A (BFA) and tunicamycin (TM). (A) Relative changes in extracellular GLuc-SERCaMP activity from SH-SY5Y-GLuc-SERCaMP stable cells treated with TM (0–10 μg/mL; black X axis) or BFA (0-750nM; gray X axis) for 8 hrs. GLuc activity was measured using coelenterazine plus NLuc inhibitor (mean ± SEM, n = 4 wells/transfection/ treatment). As previously observed [9], TM and BFA do not increase the extracellular levels GLuc-SERCaMP. (B) Relative changes in the extracellular (left Y axis, solid lines) and intracellular (right Y axis, dotted lines) NLuc levels using furimazine as the substrate. SH-SY5Y-GLuc-SERCaMP stable cells were transfected with 5X-UPRE-secNLuc and treated with TM (0–10 μg/mL; black X axis) or BFA (0-750nM; gray X axis) for 8 hrs then assayed for NLuc activity (mean ± SEM, n = 4 wells/transfection/ treatment, #p<0.05, ## or **p<0.01, ### or ***p<0.001, #### or ****p<0.0001 1-way ANOVA Dunnett’s test vs vehicle). There is small increase in extracellular NLuc activity (1.2 fold increase) at the highest concentration of TM (10 μg/mL) and BFA causes a slight decrease in extracellular NLuc activity. In contrast, the intracellular levels of NLuc are increased ~5 fold and ~30 fold for TM and BFA, respectively, **indicating a strong activation of the 5X-UPRE-secNLuc.**
(PDF)

S5 Fig Raw data for Figs 1–4 in manuscript. Tabs are labeled to match figure panels.
(XLSX)

S6 Fig Raw data for supplemental S1–S4 Figs. Tabs are labeled to match figure panels.
(XLSX)

S1 Table Sequences of PCR primers and probes. (PDF)
