# Supplementary material for: Longitudinal monitoring of Gaussia and Nano luciferase activities to concurrently assess ER calcium homeostasis and ER stress in vivo
Source: PLoS One. 2017 Apr 12;12(4):e0175481. doi: 10.1371/journal.pone.0175481 (PMC5389830; doi:10.1371/journal.pone.0175481)
Supplement: S1 Fig — A) secNLuc and GLuc both show activity with coelenterazine substrate. Culture medium was collected from SH-SY5Y cells transfected with secNLuc or GLuc after 48 hrs. Light emission was monitored over 10 min after adding 8μM coelenterazine (mean ± SD, n = 2). (B) Kinetic analysis of secNLuc and GLuc light emission using 8μM coelenterazine containing 5μM NLuc inhibitor (mean ± SD, n = 2) (C) Kinetic analysis of secNLuc and GLuc light emission with 1X furimazine substrate (mean ± SD, n = 2). (D) Linear relationship is observed for secNLuc concentration versus light emission for 0.05X furimazine substrate. Medium was collected from SH-SY5Y cells transfected with secNLuc and diluted with medium from untransfected SH-SY5Y cells. Light emission was measured (mean ± SD, n = 4). (E) Linear relationship is observed for GLuc concentration versus light emission using coelenterazine substrate (8μM). Medium was collect emission using coelenterazine substrate (8μM). Medium was collected from SH-SY5Y cells transfected with GLuc and diluted with medium from untransfected SH-SY5Y cells. Light emission was measured (mean ± SD, n = 3) (F) NLuc inhibitor has activity on secNLuc, but not GLuc in plasma. NLuc inhibitor at various concentrations was added to coelenterazine substrate (100μM final) and light emission was measured in plasma containing GLuc-SERCaMP or secNLuc (mean ± SD, n = 3). elenterazine substrate (100μM final) and light emission was measured in plasma containing GLuc-SERCaMP or secNLuc (mean ± SD, n = 3). (PDF) [file pone.0175481.s001.pdf]

# Supplemental Figure 1- Further characterization of the measurement of dual luciferases

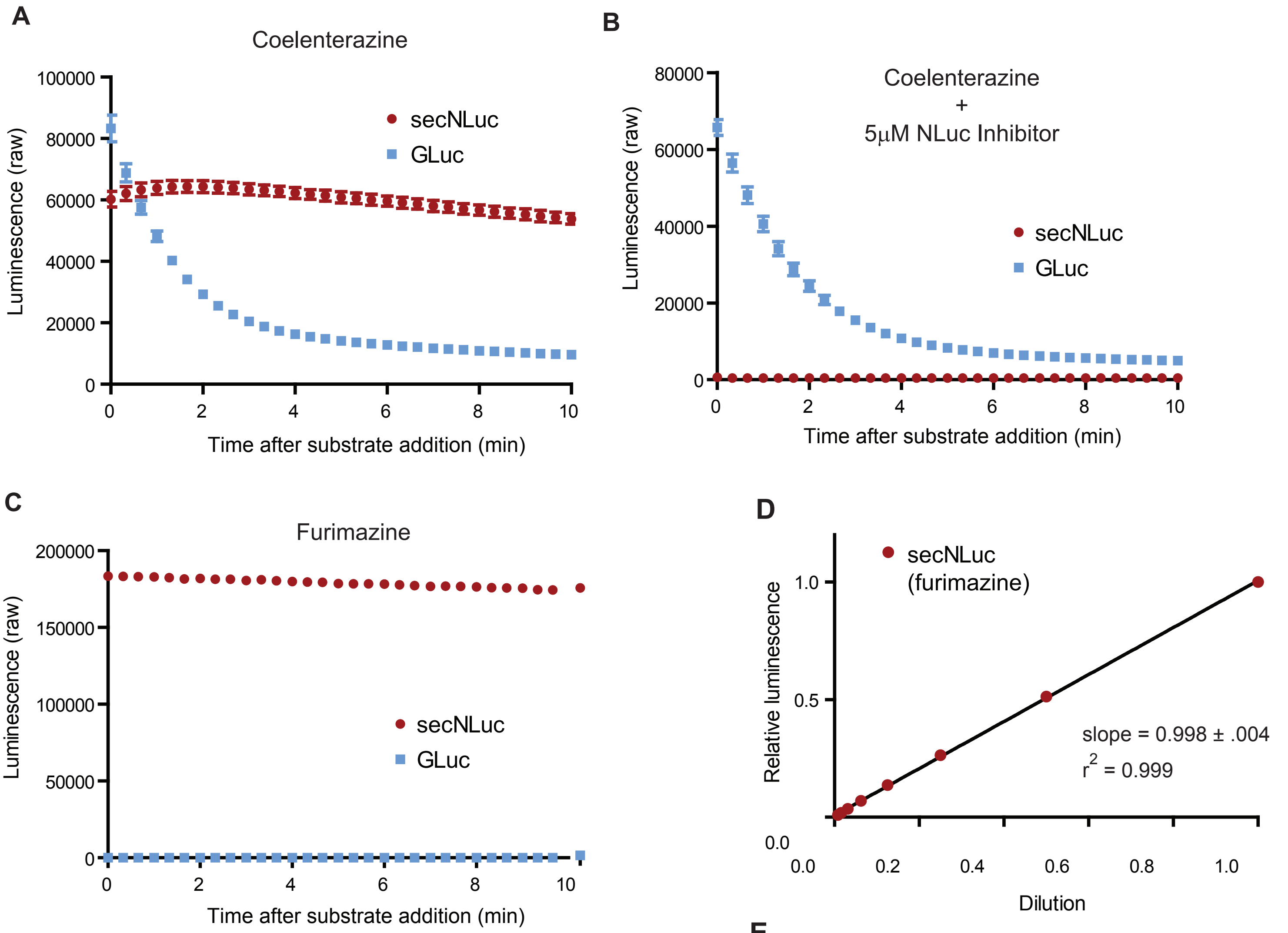


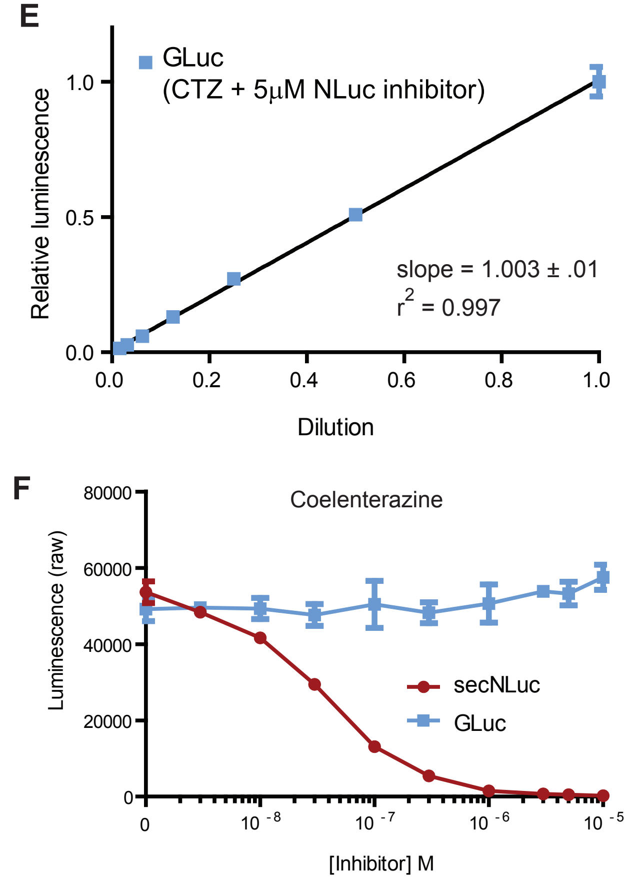


**Supplemental Figure S1: Development of approach to measure GLuc and secNLuc from biological samples containing both proteins.** (A) secNLuc and GLuc both show activity with coelenterazine substrate. Culture medium was collected from SH-SY5Y cells transfected with secNLuc or GLuc after 48 hrs. Light emission was monitored over 10 min after adding 8μM coelenterazine (mean ± SD, n=2). (B) Kinetic analysis of secNLuc and GLuc light emission using 8μM coelenterazine containing 5μM NLuc inhibitor (mean ± SD, n=2) (C) Kinetic analysis of secNLuc and GLuc light emission with 1X furimazine substrate (mean ± SD, n=2). (D) Linear relationship is observed for secNLuc concentration versus light emission for 0.05X furimazine substrate. Medium was collected from SH-SY5Y cells transfected with secNLuc and diluted with medium from untransfected SH-SY5Y cells. Light emission was measured (mean ± SD, n=4). (E) Linear relationship is observed for GLuc concentration versus light emission using coelenterazine substrate (8μM). Medium was collected from SH-SY5Y cells transfected with GLuc and diluted with medium from untransfected SH-SY5Y cells. Light emission was measured (mean ± SD, n=3) (F) NLuc inhibitor has activity on secNLuc, but not GLuc in plasma. NLuc inhibitor at various concentrations was added to coelenterazine substrate (100μM final) and light emission was measured in plasma containing GLuc-SERCaMP or secNLuc (mean ± SD, n=3).
