# Supplementary material for: Longitudinal monitoring of Gaussia and Nano luciferase activities to concurrently assess ER calcium homeostasis and ER stress in vivo
Source: PLoS One. 2017 Apr 12;12(4):e0175481. doi: 10.1371/journal.pone.0175481 (PMC5389830; doi:10.1371/journal.pone.0175481)
Supplement: S2 Fig — Medium was collected from SH-SY5Y cells transiently transfected with scAAV-CMV-secNLuc plasmid. 100 μl of furimazine substrate, diluted as indicated in the figure inset, was added to 5μl of culture medium and luminescence was measured every 20 sec over 20 min. (A) Raw luminescence values (mean ± SD, n = 3). (B) Luminescence values were normalized to time zero to assess decay kinetics over the time course (mean ± SD, n = 3). (PDF) [file pone.0175481.s002.pdf]

Supplemental Figure 2- Dilution of furimazine

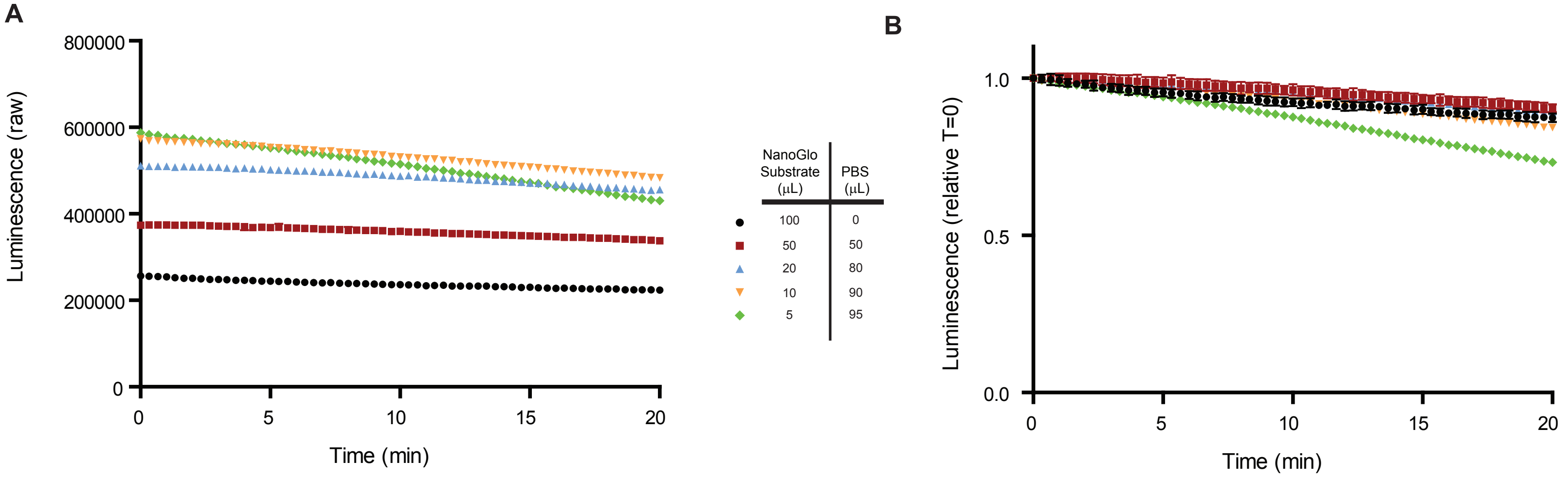

Supplemental Figure S2: Diluting furimazine substrate in PBS provides sufficient signal and acceptable light emission kinetics. Medium was collected from SH-SY5Y cells transiently transfected with scAAV-CMV-secNLuc plasmid. 100 µl of furimazine substrate, diluted as indicated in the figure inset, was added to 5µl of culture medium and luminescence was measured every 20 sec over 20 min. (A) Raw luminescence values (mean ± SD, n=3). (B) Luminescence values were normalized to time zero to assess decay kinetics over the timecourse (mean ± SD, n=3).
