# Supplementary material for: Longitudinal monitoring of Gaussia and Nano luciferase activities to concurrently assess ER calcium homeostasis and ER stress in vivo
Source: PLoS One. 2017 Apr 12;12(4):e0175481. doi: 10.1371/journal.pone.0175481 (PMC5389830; doi:10.1371/journal.pone.0175481)
Supplement: S3 Fig — (A) SH-SY5Y cells transfected with 5x-UPRE-secNLuc or MinP-secNLuc and treated with Tg (0-300nM) for 24 hrs (mean ± SD, n = 12 wells/Tg treatment, ****p<0.0001, 2-way ANOVA).(B) ATP viability of SH-SY5Y cells transfected with 5X-UPRE-secNLuc or MinP-secNLuc and treated with Tg (0-300nM) for 24 hrs (mean ± SD, n = 12 wells/Tg treatment) (C) SY5Y cells transfected with 5X-UPRE-secNLuc or MinP-secNLuc and treated with 100 nM thapsigargin for indicated time (mean ± SEM, n = 6 wells/timepoint ***p<0.001, ****p<0.0001, 2-way ANOVA, Sidak’s multiple comparison test). D) Relative expression of BiP and ERdj4 after 16 hrs activation of HFR.ATF6 with 10 μM trimethoprim (TMP) and/or XBP1 with1 μg/mL doxycycline (DOX) (mean ± upper and lower limits (2^-ΔΔCt±SD), n = 6, ****p<0.0001 as compared to control, ####p<0.0001 as compared to DOX only, %%%p<0.001 as compared to TMP only, one-way ANOVA, Tukey’s multiple comparison test). (PDF) [file pone.0175481.s003.pdf]

Supplemental Figure 3- Further characterization of secNLuc constructs

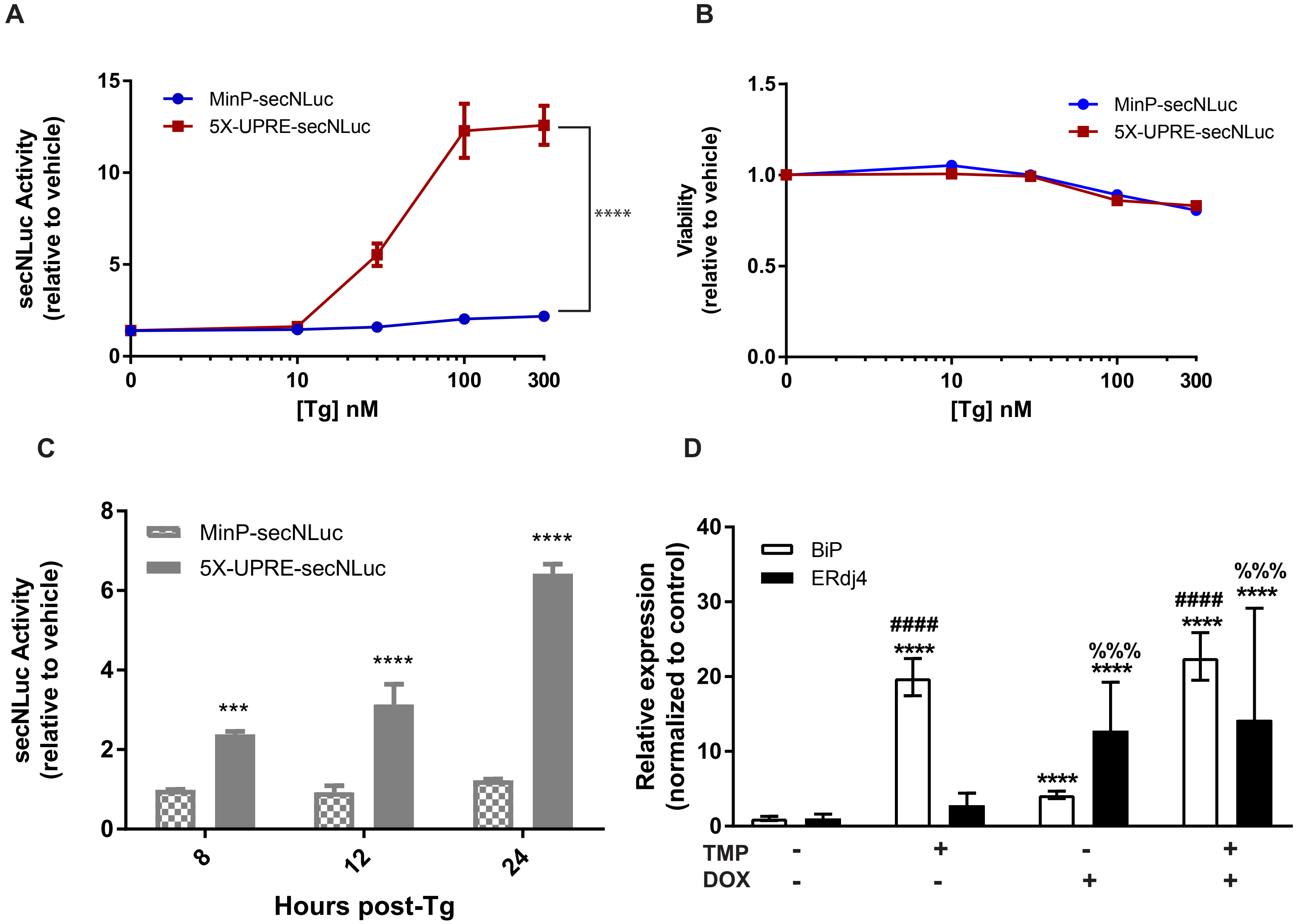

Supplemental Figure S3: Further characterization of 5X-UPRE-secNLuc constructs. (A) SH-SY5Y cells transfected with 5x-UPRE-secNLuc or MinP-secNLuc and treated with Tg (0-300nM) for 24 hrs (mean ± SD, n=12 wells/Tg treatment, ****p<0.0001, 2-way ANOVA).(B) ATP viability of SH-SY5Y cells transfected with 5X-UPRE-secNLuc or MinP-secNLuc and treated with Tg (0-300nM) for 24 hrs (mean ± SD, n=12 wells/Tg treatment) (C) SY5Y cells transfected with 5X-UP-RE-secNLuc or MinP-secNLuc and treated with 100 nM thapsigargin for indicated time (mean ± SEM, n=6 wells/timepoint ***p<0.001, ****p<0.0001, 2-way ANOVA, Sidak's multiple comparison test). D) Relative expression of BiP and ERdj4 after 16 hrs activation of DHFR.ATF6 with 10 μM trimethoprim (TMP) and/or XBP1 with1 μg/mL doxycycline (DOX) (mean ± upper and lower limits (2^-ΔΔCt±SD), n=6, ****p<0.0001 as compared to control, ####p<0.0001 as compared to DOX only, %%%p<0.001 as compared to TMP only, one-way ANOVA, Tukey's multiple comparison test).
