# Supplementary material for: Longitudinal monitoring of Gaussia and Nano luciferase activities to concurrently assess ER calcium homeostasis and ER stress in vivo
Source: PLoS One. 2017 Apr 12;12(4):e0175481. doi: 10.1371/journal.pone.0175481 (PMC5389830; doi:10.1371/journal.pone.0175481)
Supplement: S4 Fig — (A) Relative changes in extracellular GLuc-SERCaMP activity from SH-SY5Y-GLuc-SERCaMP stable cells treated with TM (0–10 μg/mL; black X axis) or BFA (0-750nM; gray X axis) for 8 hrs. GLuc activity was measured using coelenterazine plus NLuc inhibitor (mean ± SEM, n = 4 wells/transfection/ treatment). As previously observed [9], TM and BFA do not increase the extracellular levels GLuc-SERCaMP. (B) Relative changes in the extracellular (left Y axis, solid lines) and intracellular (right Y axis, dotted lines) NLuc levels using furimazine as the substrate. SH-SY5Y-GLuc-SERCaMP stable cells were transfected with 5X-UPRE-secNLuc and treated with TM (0–10 μg/mL; black X axis) or BFA (0-750nM; gray X axis) for 8 hrs then assayed for NLuc activity (mean ± SEM, n = 4 wells/transfection/ treatment, #p<0.05, ## or **p<0.01, ### or ***p<0.001, #### or ****p<0.0001 1-way ANOVA Dunnett’s test vs vehicle). There is small increase in extracellular NLuc activity (1.2 fold increase) at the highest concentration of TM (10 μg/mL) and BFA causes a slight decrease in extracellular NLuc activity. In contrast, the intracellular levels of NLuc are increased ~5 fold and ~30 fold for TM and BFA, respectively, indicating a strong activation of the 5X-UPRE-secNLuc. (PDF) [file pone.0175481.s004.pdf]

Supplemental Figure 4- Dual luciferase assay using TM and BFA

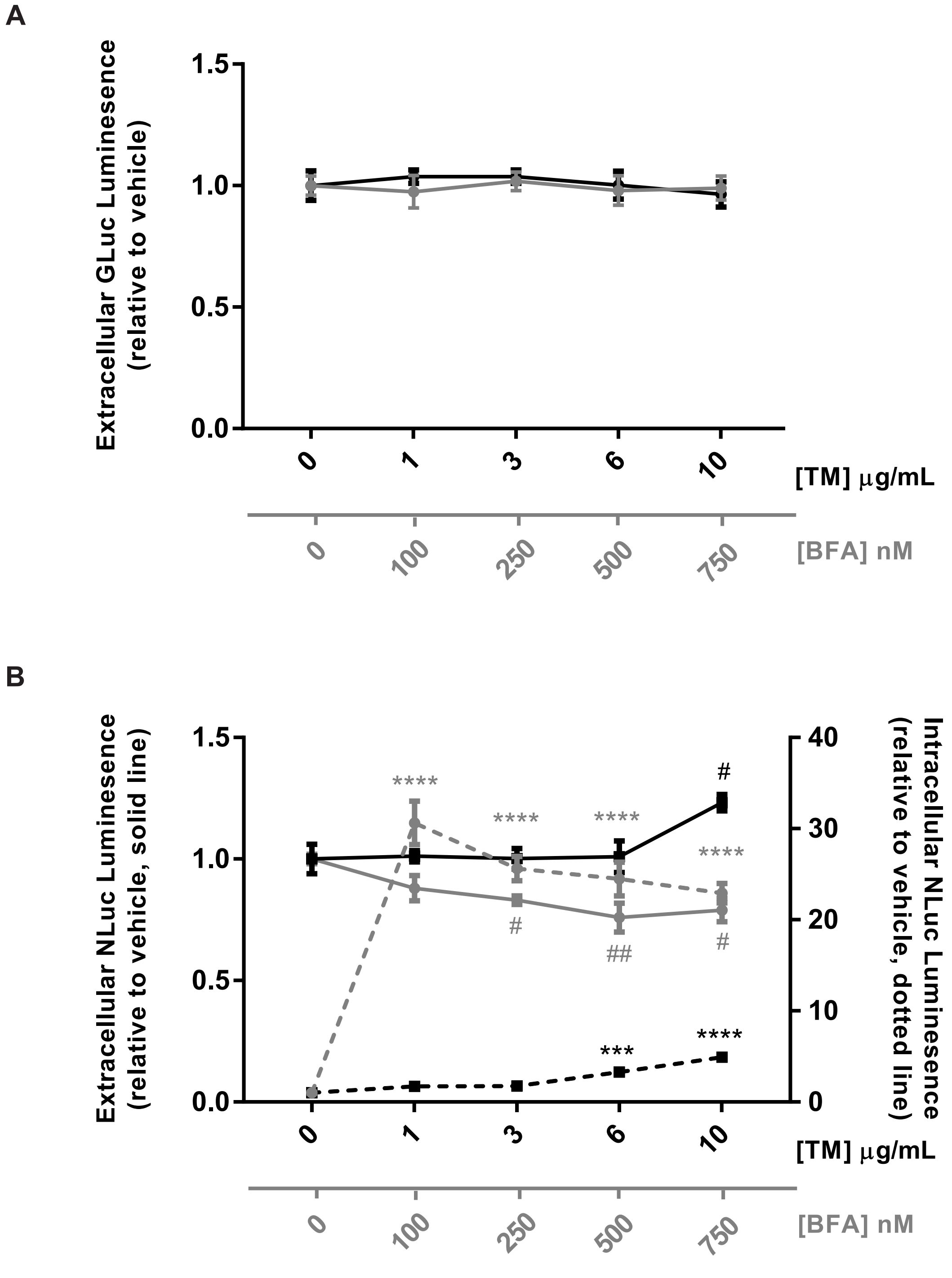

Supplemental Figure S4: Dual luciferase assay using brefeldin A (BFA) and tunicamycin (TM). (A) Relative changes in extracellular GLuc-SERCaMP activity from SH-SY5Y-GLuc-SERCaMP stable cells treated with TM (0-10 µg/mL; black X axis) or BFA (0-750nM; gray X axis) for 8 hrs. GLuc activity was measured using coelenterazine plus NLuc inhibitor (mean ± SEM, n=4 wells/transfection/ treatment). As previously observed [9], TM and BFA do not increase the extracellular levels GLuc-SERCaMP. (B) Relative changes in the extracellular (left Y axis, solid lines) and intracellular (right Y axis, dotted lines) NLuc levels using furimazine as the substrate. SH-SY5Y-GLuc-SERCaMP stable cells were transfected with 5X-UP-RE-secNLuc and treated with TM (0-10 µg/mL; black X axis) or BFA (0-750nM; gray X axis) for 8 hrs then assayed for NLuc activity (mean ± SEM, n=4 wells/transfection/ treatment, #p<0.05, ## or **p<0.01, ### or ***p<0.001, #### or ****p<0.0001 1-way ANOVA Dunnett's test vs vehicle). There is small increase in extracellular NLuc activity (1.2 fold increase) at the highest concentration of TM (10 µg/mL) and BFA causes a slight decrease in extracellular NLuc activity. In contrast, the intracellular levels of NLuc are increased ~5 fold and ~30 fold for TM and BFA, respectively, indicating a strong activation of the 5X-UPRE-secNLuc.
