# Supplementary material for: Longitudinal monitoring of Gaussia and Nano luciferase activities to concurrently assess ER calcium homeostasis and ER stress in vivo
Source: PLoS One. 2017 Apr 12;12(4):e0175481. doi: 10.1371/journal.pone.0175481 (PMC5389830; doi:10.1371/journal.pone.0175481)
Supplement: S1 Table — (PDF) [file pone.0175481.s007.pdf]

Supplemental Table 1

Name	Sequence 5'-3'
ASNSF (human, rat)	ggattggctgccttttatcagg
ASNSR (human)	ggcttctttcagctgcttcaac
ASNSR (rat)	ctccttgagttgcttcagcag
ASNS probe (human, rat) (FAM, BHQ1)	tggactccagcttggttgctgcc
ERdj4F (human, rat)	gccatgaagtaccaccctg
ERdj4R (human)	ccactagtaaaagcactgtgtc
ERdj4R (rat)	ccattagtaaaagcactgtgtcc
ERdj4 probe (human, rat) (FAM, BHQ1)	ctgcaatctctctgaattttgcttcagc
Grp78F (human)	gttgtggccactaatggagatac
Grp78R (human)	ggagtttctgcacagctctattg
Grp78 probe (human) (FAM, BHQ1)	acgctggtcaaagtcttctccaccca
Grp78F (rat)	agtggtggccactaatggagac
Grp78R (rat)	ggagtttctgcacagctctgttg
Grp78 probe (rat) (FAM, BHQ1)	cccgctgatcaaagtcttccccacccag
Hyou1F (rat)	gcagacctgttggcactgag
Hyou1R (rat)	caccggagttttcctccgaga
Hyou1 probe (rat) (FAM, BHQ1)	ccaggtccacagacatcacagccagtgt
Ube2iF (human)	gtgtgcctgtccatcttagag
Ube2iR (human)	gctgggtcttggatatttggttc
Ube2i probe (human) (FAM, BHQ1)	caaggactggaggccagccatcac
Ube2iF (rat)	gccaccactgtttcatccaaa
Ube2iR (rat)	gccgccagtccttgtcttc
Ube2i probe (rat) (FAM, BHQ1)	cgtgtatccttctggcacagtgtgc
